# A Mother’s Story, Mitogenome Relationships in the Genus *Rupicapra*

**DOI:** 10.3390/ani11041065

**Published:** 2021-04-09

**Authors:** Laura Iacolina, Elena Buzan, Toni Safner, Nino Bašić, Urska Geric, Toni Tesija, Peter Lazar, María Cruz Arnal, Jianhai Chen, Jianlin Han, Nikica Šprem

**Affiliations:** 1 Faculty of Agriculture, University of Zagreb, 10000 Zagreb, Croatia; lauraiacolina@gmail.com (L.I.); ttesija@agr.hr (T.T.); nsprem@agr.hr (N.Š.); 2 Faculty of Mathematics, Natural Sciences and Information Technologies, University of Primorska, 6000 Koper, Slovenia; elena.buzan@upr.si (E.B.); nino.basic@famnit.upr.si (N.B.); urska.geric@famnit.upr.si (U.G.); 3 Department of Chemistry and Bioscience, Aalborg University, 9220 Aalborg, Denmark; 4 Environmental Protection College, 3320 Velenje, Slovenia; 5 Centre of Excellence for Biodiversity and Molecular Plant Breeding (CoE CroP-BioDiv), 1000 Zagreb, Croatia; 6 Andrej Marušič Institute, University of Primorska, 6000 Koper, Slovenia; 7 Institute of Mathematics, Physics, and Mechanics, 1000 Ljubljana, Slovenia; 8 Department of Breeding and Diseases of Game, Fish and Bees, Ecology and Cynology, University of Veterinary Medicine and Pharmacy, Komenského 73, 04181 Košice, Slovakia; peter.lazar@uvlf.sk; 9 Facultad de Veterinaria, Universidad de Zaragoza, 50013 Zaragoza, Spain; maricruz@unizar.es; 10 Institutes for Systems Genetics, West China Second University Hospital, Sichuan University, Chengdu 610041, China; jianhaichen@scu.edu.cn; 11 Institute of Animal Science, Chinese Academy of Agricultural Sciences (CAAS), Beijing 100193, China; H.JIANLIN@cgiar.org

**Keywords:** chamois, mitogenome, phylogeny, *Rupicapra*

## Abstract

**Simple Summary:**

Two species of chamois (*Rupicapra rupicapra* and *R. pyrenaica*) are currently recognized by taxonomy and further subdivided into seven and three subspecies, respectively. However, recent research based on molecular markers finds this classification questionable. We aim to increase the resolution of published research on chamois phylogeny by including mitogenomes of all available subspecies, including the previously unpublished mitogenomes of *R. r. balcanica* and *R. r. tatrica* subspecies. The inferred phylogeny based on the full mitogenomes confirms the previously reported genus subdivision in three clades and its monophyletic positioning within the *Caprinae.* Phylogeny and taxonomy of *Rupicapra* species thus remain controversial prompting for the inclusion of archeological remains to solve the controversy.

**Abstract:**

Although the two species of chamois (*Rupicapra rupicapra* and *R. pyrenaica*) are currently classified as least-concern by the IUCN (International Union for Conservation of Nature), inconsistencies on the subspecies classification reported in literature make it challenging to assess the conservation status of the single subspecies. Previous studies relying on mitochondrial genes, sometimes in combination with nuclear or Y-chromosome markers, reported the presence of clusters corresponding to the geographic distribution but highlighting ambiguities in the genus phylogeny. Here we report novel de novo assembled sequences of the mitochondrial genome from nine individuals, including previously unpublished *R. r. balcanica* and *R. r. tatrica* subspecies, and use them to untangle the genus phylogeny. Our results based on the full mitogenome inferred phylogeny confirm the previously reported genus subdivision in three clades and its monophyletic positioning within the *Caprinae.* Phylogeny and taxonomy of *Rupicapra* species thus remain controversial prompting for the inclusion of archeological remains to solve the controversy.

## 1. Introduction

The chamois, genus *Rupicapra*, is the most abundant mountain-dwelling ungulate in Europe and the Near East, and is currently recognized to be divided into two species: *Rupicapra rupicapra* (Northern chamois) and *Rupicapra pyrenaica* (Southern chamois), further subdivided into seven (*cartusiana*, *rupicapra*, *balcanica*, *tatrica*, *carpatica*, *caucasica*, and *asiatica*) and three (*parva*, *pyrenaica*, and *ornata*) subspecies, respectively [1,2]. The conservation status of the species requires consideration. Neither of the species is threatened and both species are currently classified as least-concern in the IUCN Red List of Threatened Species [3,4]. Although some subspecies are protected at the national level in (part of) their distribution, detailed information on the conservation status of the different subspecies is patchy, if any, and chamois may be one of the most threatened European ungulates if considered at the subspecies level [2]. This picture is further complicated by molecular controversy concerning the subspecies subdivision based on morphological and behavioral characters. While some markers provided some support for this classification mitochondrial DNA (mtDNA) identified nominal species as paraphyletic [2,5] and references therein.

Previous studies relying on cytochrome *b* (*cytb*) identified three lineages geographically separated, a western clade in the Western Alps and Iberia, a central clade in the Apennines and the Chartreuse Massif, and an eastern clade covering all regions east of the Alps [6]. This subdivision, later confirmed by a combination of mitochondrial regions [7], differs from the pattern observed with microsatellites, mitochondrial pseudogenes, and introns [7,8,9]. The analyses using nuclear markers, although recognizing geographically structured clades, more closely resemble the subspecies subdivision based on morphological characteristics and highlight a complex population history strongly shaped by male-biased dispersal [7,8,9], as confirmed by Y-chromosome lineages [5]. However, mitochondrial genes evolution rates differ [10,11,12], with mutations accumulating faster in non-coding than in coding regions, and are strongly influenced by energetic demands [12,13,14], thus using the whole mitochondrial genome (mitogenome) could help to address different phylogenetic aspects [15,16]. So far, very few *Rupicapra* mitogenomes are available. Hassanin et al. [17] were the first to use the potential of mitogenomes to investigate phylogenetic relationships within the *Caprini* tribe but were unable to resolve the relationships of *Rupicapra* and a few other genera. In their study, the two *Rupicapra* species formed a separate branch in the tree [17] but represented only two of the three mitochondrial clades. The later addition of two mitogenomes of subspecies previously attributed to the central clade [18] confirmed the subdivision in three clades and the non-concordance with taxonomic classification.

To further complicate the picture, reintroductions and translocations—if performed without previous knowledge on the involved individuals’ taxonomic status [19,20]—might introduce an additional confounding effect. One of these examples is in the Northern Dinaric mountains, Mt. Velebit (Croatia), where *R. r. rupicapra* and *R. r. balcanica* were introduced for hunting purposes and led to a molecularly identified hybrid population [21]. However, such events might be difficult to trace due to a lack of historic records or illegal practices, thus leading to incorrect assumptions on the (sub)species being sampled and hampering phylogenetic and taxonomic reconstructions.

In this study, we aimed to contribute to the unraveling of the complex genus phylogeny by increasing the number of individuals and subspecies investigated using the whole mitochondrial genome. Our results confirmed the grouping of individuals from Mt. Velebit with the Balkan subspecies, the subdivision of the genus into three mitochondrial clades and its monophyletic positioning within the *Caprini*, in a sister-group relationship with *Ammotragus* and *Arabitragus*, while prompting for additional multi-marker studies also involving historical samples to disentangle the genus phylogeny and taxonomy.

## 2. Materials and Methods

### 2.1. Sampling and DNA Sequencing

We collected samples from *Rupicapra* subspecies (*rupicapra n* = 4, *balcanica n* = 2, *tatrica n* = 2, *balcanica* x *rupicapra* putative hybrids *n* = 2, and *pyrenaica n* = 2). All samples were collected from dead animals during regular hunting seasons. Genomic DNA was extracted from tissue samples using a standard phenol-chloroform method. DNA fragments were then treated according to the Illumina DNA sample preparation protocol. Genome sequencing was performed on the Illumina HiSeq 2500 platform with paired-end adaptors and 100 bp reads length for libraries of 350 bp inserts. To investigate the phylogenetic relationships, we downloaded one mitogenome sequence for each of the 34 (sub)species from the subfamily *Caprinae* available in GenBank and five outgroups: *Damaliscus pygarus*, *Damaliscus lunatus*, *Bos taurus*, *Bubalus bubalis*, and *Muntiacus reevesi* (Table S1).

### 2.2. Genome Assembly and Validation

Raw sequences were corrected using the Pollux program [22] with default settings. Mitochondrial DNA, due to the presence of multiple copies per cell, had higher coverage and depth in whole-genome sequences which, if results are of sufficient quality, allows for de novo assembly. After screening with Pollux, we excluded two samples that did not pass the quality filtering. To avoid ambiguity of the assembled sequences, we used two approaches: a de novo assembly followed by an assembly relying on a reference genome. MitoZ [23] was used for the de novo approach with default settings. We then used NOVOPlasty [24] “seed-and-extend” method using the GenBank *R. rupicapra* sequence (FY207539) as seed. The resulting circular genomes were verified as belonging to the Genus in BLAST [25]. Obtained mitochondrial sequences were further validated by aligning them against GenBank *R. rupicapra* sequence using MEGA X [26], Bioedit [27], and BWA [28] software. Visualization of results was done using GenomeVx software [29].

### 2.3. Alignment, Post Processing, and Annotation of Protein Coding Genes

Newly assembled mitogenomes that passed the quality controls (Table 1) were aligned in Mega X [26] using Clustal W [30] considering the algorithm computational demand and the size of our file [31] and checked by eye. Downloaded sequences were also visually inspected for consistency and exclude the possible presence of erroneous rearrangements and nuclear pseudogenes [8,17,31]. To avoid erroneous hypothesis on homology, all indels, positions with ambiguity in the position of the gaps, and the portion of the control region presenting tandem repeats in the *Caprini* [17] were excluded. The final alignment consisted of 15,383 bp and 40 sequences. GeSeq [32] was used to identify both protein-coding and tRNA, with the embedded tRNAscan-SE v2.0.5 [33], regions setting the sequence source to linear mtDNA. DNAsp v6.12.03 [34] was used to infer the number of haplotypes, haplotype (h) and nucleotide (π) diversity, number of polymorphic sites (S), GC content, and Fu’s (Fs) statistic, for both the whole mitogenome and each individual protein-coding region for each *Rupicapra* subspecies. The ND6 region was reverse complemented to present the same reading direction as the other protein-coding sequences [35]. DNAsp was additionally used to identify the number of synonymous (SS) and non-synonymous (NSS) sites and to compute the ratio between synonymous and non-synonymous substitutions. The overall mean distance for synonymous substitutions per synonymous site (pS) and non-synonymous substitutions per non-synonymous site (pN) were calculated using Mega X according to the Nei-Gojobori method [36] with 1000 bootstraps.

### 2.4. Phylogenetic Analyses

Phylogenetic relationships among *Caprinae* species were reconstructed using a reduced alignment containing a single sequence per *Rupicapra* subspecies, mitogenome sequences of *Caprinae* species available in GenBank, and five *Bovidae* as outgroups. The complete dataset used for this analysis consisted of 45 mitogenome sequences. Relationships within *Rupicapra* were investigated using all *Rupicapra* sequences available in GenBank (*n* = 5) and the nine new sequences obtained within this research. Mitogenome sequences of *Ammotragus lervia* and *Arabitragus jakari* were included as outgroups. PartitionFinder2 [37] was used to infer partitioning and corresponding evolutionary models, whereas ModelFinder in IQ-TREE [38] was used to select the most accurate evolutionary model for the complete mitogenome. Since partitioning the sequence did not lead to differences in tree topology (data not shown) we herewith specify evolutionary models only for the whole sequence. Two phylogenetic approaches were applied for tree inference: the maximum likelihood method in IQ-TREE [39] and Bayesian inference in MrBayes 3.2.7 [40]. Stochastic inference on both *Caprinae* and *Rupicapra* datasets was computed with IQ-TREE default parameters, genetic code set to vertebrate mitochondrial, substitution model TIM2+I+G4, and ultrafast bootstrap analysis UFBoot2 [41].

Bayesian inference on the *Caprinae* was performed with GTR+I+G4 model and Markov chain Monte Carlo simulation for 1.2 million generations. The trees were sampled every 500 generations until reaching an average standard deviation of split frequencies of 0.011. MrBayes parameters for *Rupicapra* were GTR+G4 and 1.7 million generations, sampled every 200 generations until reaching an average standard deviation of split frequencies of 0.006. For both analyses, the convergence of chains was additionally checked by verifying the stationarity of the log probability graphs [42]. For each run, 25% of the initial trees were discarded as burn-in.

Tree editing and visualization were performed using the R package ggtree [43]. Within and between group genetic distances of *Rupicapra* subspecies were calculated in Mega X using the Maximum Composite Likelihood model [44], rate variation among sites modeled with an uneven gamma distribution (shape parameter = 1) [45] and including all codon positions.

## 3. Results

### 3.1. Diversity of Rupicapra Mitogenome

After quality control screening, we obtained nine newly sequenced complete mitogenomes of *Rupicapra*, which showed the typical organization (Figure S1) of mammalian mitochondrial genomes, incomplete stop codons, overlapping coding regions, and different start codons [46,47]. Results of all methods used for validation of the alignment were concordant. The alignment with the five already available conspecific sequences and 39 from other species had an initial length of 16,850 bp and, after removal of sites with ambiguities, gaps, and the tandem repeat of the control region, the final alignment was 15,383 bp and presented 6075 variable sites (39.49%), out of which 4914 (31.94%) were parsimony informative and 1161 (7.55%) were singletons.

**Figure 1:**
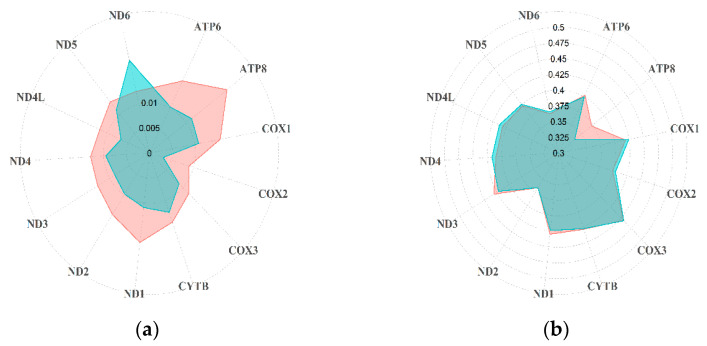
Differences between *R. rupicapra* (green) and *R. pyrenaica* (red) in terms of (**a**) nucleotide diversity and (**b**) GC content.

The 14 *Rupicapra* sequences included in the full dataset showed an overall high haplotype (h = 0.945; SD = 0.045) and nucleotide (π = 0.019; sd = 0.003) diversity, with high variability between the two species and among subspecies (Table 1). A similarly high variability between *R. rupicapra* and *R. pyrenaica* was observed in the variability of the coding regions, with *R. rupicapra* showing in general lower nucleotide and haplotype diversity, despite the larger sample size, with some exceptions (Figure 1a and Table S2). However, the GC content and the ratio between synonymous and non-synonymous substitutions were overall concordant between the two species (Figure 1b and Table S2). Although the number of NSS was higher than the SS (Table S2), the pS-pN difference showed an overall higher proportion of pS (*R. pyrenaica* 0.009, standard error –SE–0.000; *R. rupicapra* 0.008, SE 0.000).

### 3.2. Phylogenetic Relationships

**Figure 2:**
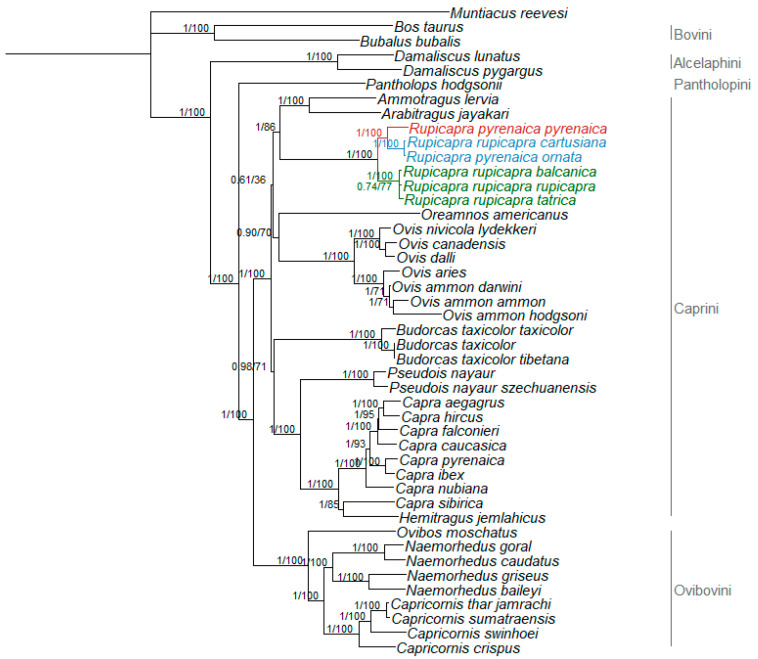
Rooted phylogenetic tree obtained by Bayesian inference for the whole *Caprinae*. Nodal supports are indicated above the nodes (posterior probability for Bayesian inference and bootstrap for maximum likelihood, respectively). Colored branches represent *Rupicapra* clades: red—clade W; blue—clade C; and green—clade E.

The Bayesian phylogenetic tree of the Caprinae dataset identified the same topology as reported by Hassanin et al. [17], with tribes Bovini and Caprini being monophyletic, sister group relationship of *Damaliscus* and the Ovicaprini, the basal divergence of *Pantholops* within the Ovicaprini, and a well-supported separation of the Ovibovini. Within the Caprini tribe, *Capra* and *Hemitragus* form a goat-like clade and the more distant *Pseudois* and *Budorcas* as sister groups; *Ovis* and *Oreamnos* group together in a sheep-like clade; *Ammotragus* and *Arabitragus* showed a sister group relationship and finally, *Rupicapra* was monophyletic (Figure 2). The maximum likelihood analysis (tree not shown) was consistent with Bayesian results, showing both the same topology and the same high support for the nodes (bootstrap values provided in Figure 2).

**Figure 3:**
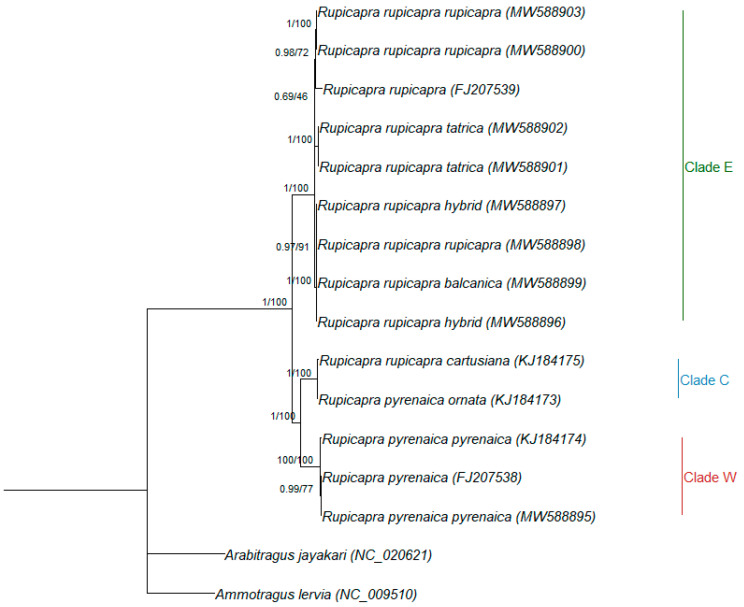
Rooted phylogenetic tree obtained by Bayesian inference for *Rupicapra* only. Nodal supports are indicated above the nodes (posterior probability for Bayesian inference and bootstrap percentages for maximum likelihood, respectively). GenBank Accession numbers are presented in parentheses.

The maximum likelihood and Bayesian analyses focusing on *Rupicapra* (Figure 3) showed the same highly supported subdivision in eastern, central, and western clades, with the western and central clade being closer (between group distance = 0.024 SE 0.002) to each other than to the eastern one (between group distance = 0.032 SE 0.003 and 0.030 SE 0.003, respectively). Within the eastern clade, *R. r. balcanica* was the most differentiated and included both sequences from the contact population from Mt. Velebit, whereas *R. r. tatrica* and *R. r. rupicapra* showed a sister group relationship (Figure 3).

## 4. Discussion

The phylogeny and taxonomy of *Rupicapra* species remain controversial despite the improvements in molecular methods over the decades and are still an intriguing question, with important implications on both the evolution and conservation of this species complex [2,9]. Initial molecular investigations using electrophoresis in the 1980s supported the subdivision into an Alpine lineage separated from the Apennine and Pyrenean one [48]. Later studies, however, revealed a much more complex phylogenetic history with contrasting nuclear, Y-chromosome, and mitochondrial patterns [5,6,7,18], whereas more recent findings provide support to the hypothesis of ancient hybridization among lineages [8,9]. Although differences among markers in phylogenetic reconstructions are not unusual, due to the evolutionary mode of each marker and the fact that they represent either matrilineal (mitochondrial), patrilineal (Y-chromosome), or biparental (nuclear) evolutionary histories, what is intriguing with *Rupicapra* is that the same marker, in this case, mitochondrial DNA, led to contrasting conclusions on the number and composition of lineages at different times, depending on the methodological approach (restriction fragment length polymorphisms or sequencing) and the region analyzed (coding, non-coding, a combination or the whole mitogenome) [2,18]. Currently, the most accepted taxonomy recognizes two species (*R. rupicapra* and *R. pyrenaica*) and three mitochondrial clades corresponding to the geographic distribution of the populations (eastern, central, and western).

To disentangle these controversies, we integrated previous knowledge on the topic with newly sequenced mitogenomes. So far only two studies used whole mitogenomes of the genus [17,18], and our results, based on a larger dataset, identified the same subdivision of the *Caprinae* tree (Figure 2) into *Pantholopini*, *Caprini,* and *Ovibovini* as previously reported [17,18], and the same eastern, central and western clades described elsewhere [5,6,7,18] for the *Rupicapra* tree (Figure 3), with the central clade being more closely related to the western than to the eastern one [18]. This observation agrees with the intermediate morphological phenotype of *R. r. cartusiana* compared to *R. pyrenaica* and *R. rupicapra* [18,49]. Additionally, concordantly with what was reported in mitogenome publications [17,50], but contrary to previous studies based on *cytb* [51,52], *Budorcas* sequences did not group with *Ovis* but were closer to *Capra* and *Pseudois*, highlighting the increased resolution provided by whole mitogenomes compared to single mitochondrial genes.

The other point worth mentioning is the presence of a sequence attributed to *R. r. rupicapra* within the *R. r. balcanica* clade. This result is particularly intriguing as it taps into an interesting management issue. The sample comes from the Northern Dinaric Mountains. The southern part of this mountain chain was identified as inhabited by a human-induced hybrid population based on the comparison between nuclear and mitochondrial markers [21]. The same study reported no shared haplotypes between the Northern and Southern Velebit populations. Although based on a single individual, our result prompts further investigation of this area to validate the extension of the contact area between the two subspecies and the potential implications for management.

The clustering of *R. r. cartusiana* sequence with *R. p. ornata* (Figure 2 and Figure 3) and its grouping in the same clade as other subspecies of *R. pyrenaica* confirms previous findings based on morphological [25] and genetic data [6,7,18], and further increases disconcordance between current chamois systematics and the results of genetic analyses.

Quite interestingly, Pérez and colleagues [18] reported that in the central clade COX genes were quite conserved while ATP genes were more variable. Our analyses at the species level observed a similar pattern but with some important differences between species. Nucleotide diversity was generally lower in *R. rupicapra* but with a trend similar to *R. pyrenaica*, with the exception of ND4L, where a slight decrease (0.007) was observed compared to the Iberian chamois (0.012), and unexpectedly high variation at the ND6 gene (0.019 and 0.013, respectively). GC content was more homogeneous between the two species, with a single noticeable difference at the ATP8 gene, with the Alpine chamois showing lower variability (0.335 and 0.372, respectively). While estimates of nucleotide diversity on small samples are found to be underestimated [53], it is interesting to notice that greater variability was observed in the species with a lower sample size, opening questions on the population history of both species. All mitochondrial genes are involved in cellular respiration and are thus extremely important for efficient energy production and aging [12,13]. However, variation in mitochondrial genes can lead to a number of pathological disorders [54,55,56]. In particular, in humans, mutations at ND genes have been associated with progressive loss of central vision [57], epilepsy [58], and muscular dystonia [59], whereas ATP8 has been associated with autoimmune skin diseases [60], epilepsy [58] and cancer [61], making the differences of variability between species worth further investigation, considering the potential implications for conservation.

## 5. Conclusions

Overall, our results provide increased resolution compared to the available literature, confirm the phylogeny previously reported for both whole and partial mitochondrial sequences and the discrepancy among markers in phylogenetic reconstruction of the genus. Pérez and colleagues [9] explained the differences among nuclear, mitochondrial, and Y-chromosome phylogenies with male-mediated introgression and female philopatry. Hybridization is a particularly controversial aspect for this genus, since both natural and human-mediated hybridization have been repeatedly hypothesized or anecdotally reported on several occasions, but confirmed on few [19]. However, the taxonomic subdivision of the genus was recently questioned again, since female philopatry might bias mitochondrial-based phylogenies and the extremely low variation of nuclear markers observed suggests a single species [9]. Further studies based on multiple markers, and possibly including archeological remains, are thus needed to solve the controversy.

## Figures and Tables

**Table 1:** Variability across the *Rupicapra* subspecies. N = number of individuals, N haplotypes = number of haplotypes, S = number of polymorphic sites, π = nucleotide diversity, h = haplotype diversity, SD = standard deviation, Fs = Fu’s Fs, TOT = total.

Subspecies	N	N Haplotypes	S	π (SD)	h (SD)	Fs	Accession Number
*R. rupicapra*	1	1					FJ207539 ^1^
*R. r. rupicapra*	3	2	52	0.002 (0.001)	0.667 (0.314)	6.481	MW588898 ^2^ MW588900 ^2^ MW588903 ^2^
*R. r. balcanica*	1	1					MW588899 ^2^
*R. r. rupicapra* x *R. r. balcanica* putative hybrid	2	2	1	0.000 (0.000)	1.000 (0.500)	0.000	MW588896 ^2^ MW588897 ^2^
*R. r. tatrica*	2	1					MW588901 ^2^ MW588902 ^2^
*R. r. cartusiana*	1	1					KJ184175 ^3^
***R. rupicapra* TOT**	**10**	**6**	**573**	**0.009 (0.004)**	**0.889 (0.075)**	**12.454**	
*R. pyrenaica*	1	1					FJ207538 ^1^
*R. p. ornata*	1	1					KJ184173 ^3^
*R. p. pyrenaica*	2	2	14	0.001 (0.000)	1.000 (0.500)	2.639	KJ184174 ^3^ MW588895 ^2^
***R. pyrenaica* TOT**	**4**	**4**	**371**	**0.012 (0.006)**	**1.000 (0.177)**	**3.426**	
**TOT**	**14**	**10**	**771**	**0.019 (0.003)**	**0.945 (0.045)**	**11.944**	

^1^ Hassanin et al. (2009); ^2^ present study; ^3^ Pérez et al. (2014).

## Data Availability

The newly assembled DNA sequences presented in this study are openly available in GenBank, with accession number of each sequence stated in the manuscript.

## Supplementary Materials

Supplementary Materials can be found at https://www.mdpi.com/article/10.3390/ani11041065/s1, Figure S1: Structural organization of *Rupicapra* mitochondrial genome; Table S1: List of sequences and accession codes; Table S2: Variability among *Rupicapra* subspecies for each gene.
