# Supplementary material for: A Mother’s Story, Mitogenome Relationships in the Genus Rupicapra
Source: Animals (Basel). 2021 Apr 9;11(4):1065. doi: 10.3390/ani11041065 (PMC8069603; doi:10.3390/ani11041065)
Supplement: Supplementary file 1 [file animals-11-01065-s001.zip › animals-1143509_SupplementaryR1.docx]

Article

A Mother’s Story, Mitogenome Relationships in the Genus
*Rupicapra*

Laura Iacolina ^1,2,3^, Elena Buzan ^2,4^, Toni Safner ^1,5,^*, Nino Bašić ^2,6,7^, Urska Geric ^2^, Toni Tesija ^1^, Peter Lazar ^8^,
María Cruz Arnal ^9^, Jianhai Chen^10^, Jianlin Han ^11^ and Nikica Šprem ^1^

**Figure S1.** Structural organization of *Rupicapra* mitochondrial genome. The color of the ring fragment indicates sequence type. Regions in the outer circle are 5’ → 3’ oriented, while those in the inner circle are reverse oriented.


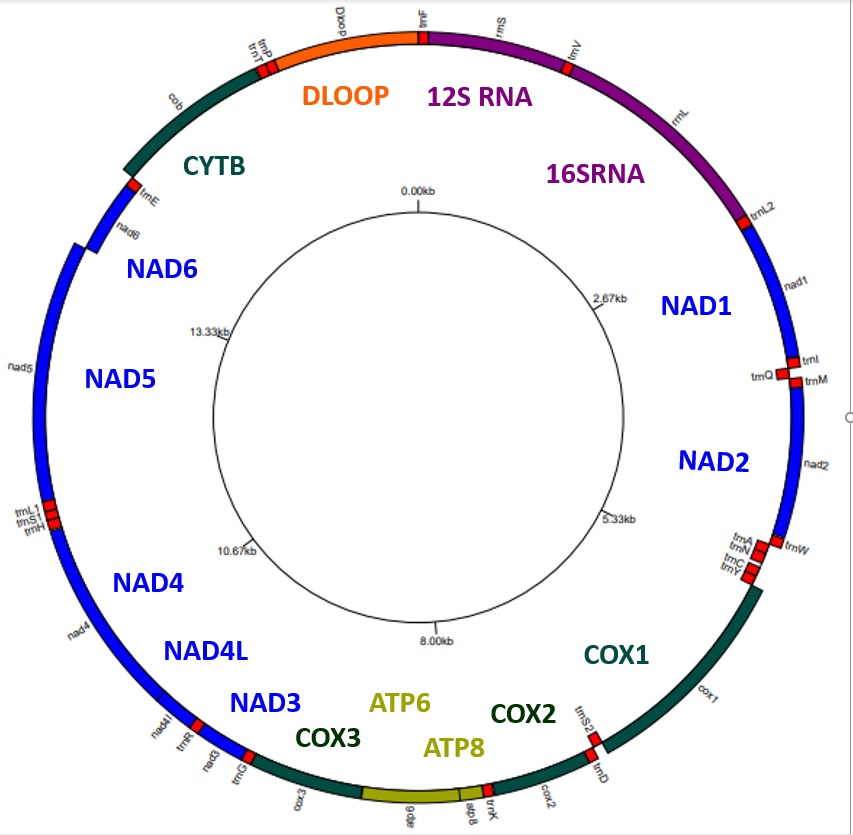


**Supplementary table S1**

**Table S1.** List of sequences and accession codes.

| Species | Subspecies | Accession code | Sampling location | Reference |
| --- | --- | --- | --- | --- |
| *Ammotragus lervia* |  | NC_009510 |  | [1] |
| *Arabitragus jayakari* |  | NC_020621 | United Arab Emirates | [2] |
| *Bos Taurus* |  | NC_006853 | Korea | Chung & Ha 2005 - direct submission |
| *Bubalus bubalis* |  | NC_049568 |  | Verma et al 2020 - direct submission |
| *Budorcas taxicolor* | *taxicolor* | NC_043930 | India, Dibang Valley | [3] |
| *Budorcas taxicolor* | *tibetana* | NC_039686 | China | [4] |
| *Budorcas taxicolor* |  | NC_013069 |  | Wu et al 2009 - direct submission |
| *Capra aegagrus* |  | KR059226 | Iran | [5] |
| *Capra caucasica* |  | NC_020683 |  | [6] |
| *Capra falconeri* |  | NC_020622 | Cytogenetic collection 2001-214, MNHN | [2] |
| *Capra hircus* |  | NC_005044 |  | [7] |
| *Capra ibex* |  | NC_020623 | Cytogenetic collection 2002-037, MNHN | [2] |
| *Capra nubiana* |  | NC_020624 | Jardin des Plantes, MNHN | [2] |
| *Capra pyrenaica* |  | NC_020625 | Spain, Sierra Nevada | [2] |
| *Capra sibirica* |  | NC_020626 | Jardin des Plantes, MNHN | [2] |
| *Capricornis crispus* |  | NC_012096 |  | Yasue et al 2009 - direct submission |
| *Capricornis sumatraensis* |  | NC_020629 | Cambodia, Phnom Tamao | [2] |
| *Capricornis swinhoei* |  | NC_010640 |  | Lee et al 2008 - direct submission |
| *Capricornis thar* | *jamrachi* | KT345703 |  | Zhang et al 2015 - direct submission |
| *Damaliscus lunatus* |  | NC_023543 |  | [8] |
| *Damaliscus pygargus* |  | NC_020627 | Vincennes zoo, MNHN | [2] |
| *Hemitragus jemlahicus* |  | NC_020628 | Cytogenetic collection 2002-005, MNHN | [2] |
| *Muntiacus reevesi* |  | NC_004069 |  | Zhang et al 2002 - direct submission |
| *Naemorhedus baileyi* |  | NC_020722 | Rotterdam Zoo | [6] |
| *Naemorhedus caudatus* |  | NC_013751 | South Korea | [9] |
| *Naemorhedus goral* |  | NC_021381 | China, Tibet, Mount Qomolangma (Everest) Nature Reserve | [10] |
| *Naemorhedus griseus* |  | NC_020723 | Thailand | [6] |
| *Oreamnos americanus* |  | NC_020630 | Cytogenetic collection 2002-547, MNHN | [2] |
| *Ovibos moschatus* |  | NC_020631 | USA, Alaska | [2] |
| *Ovis ammon* | *hodgsoni* | JX101654 | China, Tibet | [11] |
| *Ovis ammon* | *darwini* | KX609626 | China | [12] |
| *Ovis ammon* | *ammon* | MN564883 | China | [13] |
| *Ovis aries* |  | NC_001941 |  | [14] |
| *Ovis canadensis* |  | MH094035 |  | Davenport et al 2018 - direct submission |
| *Ovis dalli* |  | NC_039432 | USA | [15] |
| *Ovis nivicola* | *lydekkeri* | NC_039431 | Russia | [15] |
| *Pantholops hodgsonii* |  | NC_007441 | China, Kekexili Natural Reservation | [16] |
| *Pseudois nayaur* |  | FJ207537 | Jardin des Plantes, MNHN | [2] |
| *Pseudois nayaur* | *szechuanensis* | KP998469 | China | Liu et al 2015 - direct submission |
| *Rupicapra pyrenaica* |  | FJ207538 | France, Pyrénées | [2] |
| *Rupicapra pyrenaica* | *ornata* | KJ184173 | Italy, Apennines | [17] |
| *Rupicapra pyrenaica* | *pyrenaica* | KJ184174 | France, Pyrénées | [17] |
| *Rupicapra pyrenaica* | *pyrenaica* | MW588895 | Spain, Aragonese Pyrenees | This study |
| *Rupicapra rupicapra* |  | FJ207539 | Cytogenetic collection 2001-175, MNHN | [2] |
| *Rupicapra rupicapra* | *cartusiana* | KJ184175 | France, Chartreuse massif | [17] |
| *Rupicapra rupicapra* | *balcanica* | MW588899 | Croatia, Biokovo | This study |
| *Rupicapra rupicapra* | *rupicapra* | MW588898 | Croatia, Northern Velebit | This study |
| *Rupicapra rupicapra* | *rupicapra* | MW588900 | Croatia, Gorski Kotar | This study |
| *Rupicapra rupicapra* | *rupicapra* | MW588903 | Slovenia, Osilnica | This study |
| *Rupicapra rupicapra* | *tatrica* | MW588901 | Slovakia, National Park High Tatras | This study |
| *Rupicapra rupicapra* | *tatrica* | MW588902 | Slovakia, National Park High Tatras | This study |
| *Rupicapra rupicapra* | Suspected hybrid | MW588896 | Croatia, Southern Velebit | This study |
| *Rupicapra rupicapra* | Suspected hybrid | MW588897 | Croatia, Southern Velebit | This study |

**References**

1. Mereu, P.; Palici di Suni, M.; Manca, L.; Masala, B. Complete nucleotide mtDNA sequence of Barbary sheep (*Ammotragus lervia*). *DNA Seq.* **2008**, *19*, 241–245, doi:10.1080/10425170701550599.

2. Hassanin, A.; Ropiquet, A.; Couloux, A.; Cruaud, C. Evolution of the Mitochondrial Genome in Mammals Living at High Altitude: New Insights from a Study of the Tribe *Caprini* (*Bovidae, Antilopinae*). *J. Mol. Evol.* **2009**, *68*, 293–310, doi:10.1007/s00239-009-9208-7.

3. Kumar, A.; Gautam, K.B.; Singh, B.; Yadav, P.; Gopi, G.V.; Gupta, S.K. Sequencing and characterization of the complete mitochondrial genome of Mishmi takin (*Budorcas taxicolor taxicolor*) and comparison with the other *Caprinae* species. *Int. J. Biol. Macromol.* **2019**, *137*, 87–94, doi:10.1016/j.ijbiomac.2019.06.201.

4. Zhou, M.; Yu, J.; Li, B.; Ouyang, B.; Yang, J. The complete mitochondrial genome of *Budorcas taxicolor tibetana* (Artiodactyla: *Bovidae*) and comparison with other *Caprinae* species: Insight into the phylogeny of the genus Budorcas. *Int. J. Biol. Macromol.* **2019**, *121*, 223–232, doi:10.1016/j.ijbiomac.2018.10.020.

5. Colli, L.; Lancioni, H.; Cardinali, I.; Olivieri, A.; Capodiferro, M.R.; Pellecchia, M.; Rzepus, M.; Zamani, W.; Naderi, S.; Gandini, F.; et al. Whole mitochondrial genomes unveil the impact of domestication on goat matrilineal variability. *BMC Genomics* **2015**, *16*, doi:10.1186/s12864-015-2342-2.

6. Hassanin, A.; Delsuc, F.; Ropiquet, A.; Hammer, C.; Jansen Van Vuuren, B.; Matthee, C.; Ruiz-Garcia, M.; Catzeflis, F.; Areskoug, V.; Nguyen, T.T.; et al. Pattern and timing of diversification of Cetartiodactyla (Mammalia, Laurasiatheria), as revealed by a comprehensive analysis of mitochondrial genomes. *Comptes Rendus - Biol.* **2012**, *335*, 32–50, doi:10.1016/j.crvi.2011.11.002.

7. Hassanin, A.; Bonillo, C.; Nguyen, B.X.; Cruaud, C. Comparisons between mitochondrial genomes of domestic goat (*Capra hircus*) reveal the presence of numts and multiple sequencing errors. *Mitochondrial DNA* **2010**, *21*, 68–76, doi:10.3109/19401736.2010.490583.

8. Steiner, C.C.; Charter, S.J.; Houck, M.L.; Ryder, O.A. Molecular Phylogeny and Chromosomal Evolution of Alcelaphini (*Antilopinae*). *J. Hered.* **2014**, *105*, 324–333, doi:10.1093/jhered/esu004.

9. Jang, K.H.; Hwang, U.W. Complete mitochondrial genome of the Korean goral *Naemorhaedus caudatus* (*Ruminantia, Bovidae, Antilopinae*) and conserved domains in the control region of Caprini. *Mitochondrial DNA* **2010**, *21*, 62–64, doi:10.3109/19401736.2010.490833.

10. Yang, C.; Xiang, C.; Qi, W.; Xia, S.; Tu, F.; Zhang, X.; Moermond, T.; Yue, B. Phylogenetic analyses and improved resolution of the family Bovidae based on complete mitochondrial genomes. *Biochem. Syst. Ecol.* **2013**, *48*, 136–143, doi:10.1016/j.bse.2012.12.005.

11. Jiang, L.; Wang, G.; Tan, S.; Gong, S.; Yang, M.; Peng, Q.; Peng, R.; Zou, F. The complete mitochondrial genome sequence analysis of Tibetan argali (*Ovis ammon hodgsoni*): Implications of Tibetan argali and Gansu argali as the same subspecies. *Gene* **2013**, *521*, 24–31, doi:10.1016/j.gene.2013.03.049.

12. Mao, H.; Liu, H.; Ma, G.; Yang, Q.; Guo, X.; Lamaocao, Z. The complete mitochondrial genome of *Ovis ammon darwini* (*Artiodactyla: Bovidae*). *Conserv. Genet. Resour.* **2017**, *9*, 59–62, doi:10.1007/s12686-016-0620-1.

13. Wang, C.; Xu, H.; Li, D.; Wu, J.; Wen, A.; Xie, M.; Wang, Q.; Zhu, G.; Ni, Q.; Zhang, M.; et al. Phylogenetic and characterization of the complete mitochondrial genome relationship of Argali sheep (*Ovis ammon*). *Mitochondrial DNA Part B* **2020**, *5*, 273–274, doi:10.1080/23802359.2019.1698369.

14. Hiendleder, S.; Lewalski, H.; Wassmuth, R.; Janke, A. The complete mitochondrial DNA sequence of the domestic sheep (*Ovis aries*) and comparison with the other major ovine haplotype. *J. Mol. Evol.* **1998**, *47*, 441–448, doi:10.1007/PL00006401.

15. Dotsev, A. V; Kunz, E.; Shakhin, A. V; Petrov, S.N.; Kostyunina, O. V; Okhlopkov, I.M.; Deniskova, T.E.; Barbato, M.; Bagirov, V.A.; Medvedev, D.G.; et al. The first complete mitochondrial genomes of snow sheep (*Ovis nivicola*) and thinhorn sheep (*Ovis dalli*) and their phylogenetic implications for the genus *Ovis*. *Mitochondrial DNA Part B* **2019**, *4*, 1332–1333, doi:10.1080/23802359.2018.1535849.

16. Xu, S.-Q.; Yang, Y.-Z.; Zhou, J.; Jin, G.-E.; Chen, Y.-T.; Wang, J.; Yang, H.-M.; Wang, J.; Yu, J.; Zheng, X.-G.; et al. A Mitochondrial Genome Sequence of the Tibetan Antelope (*Pantholops hodgsonii*). *Genomics. Proteomics Bioinformatics* **2005**, *3*, 5–17, doi:10.1016/S1672-0229(05)03003-2.

17. Pérez, T.; González, I.; Essler, S.E.; Fernández, M.; Domínguez, A. The shared mitochondrial genome of *Rupicapra pyrenaica ornata* and *Rupicapra rupicapra cartusiana*: Old remains of a common past. *Mol. Phylogenet. Evol.* **2014**, *79*, 375–379, doi:https://doi.org/10.1016/j.ympev.2014.07.004.
